# Is maxillomandibular advancement an effective treatment for obstructive sleep apnea? Systematic literature review and meta-analysis

**DOI:** 10.1016/j.bjorl.2023.02.007

**Published:** 2023-03-13

**Authors:** Paulo Alceu Kiemle Trindade, Vânia dos Santos Nunes Nogueira, Silke Anna Theresa Weber

**Affiliations:** a Universidade Estadual Paulista (UNESP), Faculdade de Medicina de Botucatu, Botucatu, SP, Brazil; b Universidade de São Paulo (USP), Hospital de Reabilitação de Anomalias Craniofaciais (HRAC), Bauru, SP, Brazil

**Keywords:** OSA, Maxillomandibular advancement, Polysomnography

## Abstract

**Objectives:**

To evaluate the effectiveness of maxillomandibular advancement surgery in the treatment of Obstructive Sleep Apnea by comparing the pre- and postoperative Apnea and Hypopnea Index, in addition to classifying the degree of evidence and risk of intervention bias.

**Methods:**

A systematic review of the literature was carried out in the PUBMED, LILACS, EMBASE, SCOPUS, WEB OF SCIENCE and COCHRANE platforms, including cohort studies with polysomnographic follow-up, without other associated pharyngeal or nasal surgical procedures. The risk of study bias was assessed using the Modified Delphi technique. Pre- and postoperative Apnea and Hypopnea Index data were plotted for meta-analysis, and the quality of evidence was assessed using the GRADE system.

**Results:**

Of 1882 references, 32 articles were selected for full-text reading, of which four studies were included, totaling 83 adults with obstructive sleep apnea who underwent maxillomandibular advancement. The meta-analysis was in favor of the intervention (DM = −33.36, 95% CI −41.43 to −25.29, *p* < 0.00001), with a mean percentage reduction in the Apnea and Hypopnea Index of 79.5% after surgery, even though the level of evidence was classified as very low quality by the GRADE system.

**Conclusion:**

The meta-analysis was in favor of the intervention, characterizing maxillomandibular advancement surgery as an effective treatment for obstructive sleep apnea in adults.

## Highlights

• Maxillomandibular advancement for obstructive sleep apnea treatment.
• Volumetric alterations of the upper airways in orthognathic surgery.
• Apnea and Hypopnea Index reduction after maxillomandibular advancement.

## Introduction

Obstructive sleep apnea (OSA) is characterized by intermittent episodes of upper airway obstruction during sleep, associated with oxyhemoglobin desaturation.^1^ Physiological sleep is considered a time for recovery for the human body, preserving physical, mental and psychological health. Individuals with OSA have an altered sleep pattern and, consequently, show signs and symptoms such as fatigue, irritability, memory loss, morning headache, decreased concentration, depression and even severe systemic manifestations, such as high blood pressure, diabetes and stroke.^2^, ^3^ During sleep, reduced muscle tone predisposes to upper airway (UA) collapse. As a consequence, recurrent episodes of hypoxemia, hypercapnia, microarousals and sympathetic stimulation may occur, increasing the risk of cardiovascular, metabolic and inflammatory consequences.

The etiology is multifactorial and is usually associated with age, obesity, male gender, in addition to pathological and anatomical factors of the upper airways, which induce an increase in airflow resistance during sleep. Maxillomandibular retrusion is often associated with OSA due to the decrease in the upper airway volume resulting from this anatomical condition. The treatment of this type of dental–skeletal alteration often involves orthognathic surgery, in association with orthodontic treatment, aiming at restoring ideal dental occlusion through surgical repositioning of the maxillary bones. Maxillomandibular Advancement (MMA) has been recommended by several medical groups for the treatment of OSA, alone or in combination with complementary surgical procedures such as septoplasty, turbinectomy or uvulopalatopharyngoplasty (UPPP), with good results.^4^, ^5^, ^6^, ^7^ The positive effect of increased UA volume after MMA is already known and has been demonstrated in several studies using volumetric tomographic analysis.^8^, ^9^, ^10^

Although promising, MMA for the treatment of OSA still lacks solid evidence that correlates an improvement in polysomnographic parameters secondary to the gain in UA volume. The systematic reviews found in the medical literature do not reflect the efficiency and safety of MMA, without simultaneous combination with other pharyngeal or nasal cavity procedures, therefore, there is no analysis of the quality of the evidence of the isolated effect of MMA on OSA.

## Objectives

This systematic literature review and meta-analysis aimed to evaluate the efficacy of maxillomandibular advancement in the treatment of OSA by analyzing the apnea–hypopnea index (AHI) in pre- and postoperative polysomnography (PSG) assessments.

## Methods

### Scientific databases and study identification

This systematic review was reported in accordance with the PRISMA recommendations to identify relevant articles published in different scientific databases, such as PUBMED, LILACS, EMBASE, SCOPUS, WEB OF SCIENCE, COCHRANE and gray literature, aiming to answer whether the orthognathic surgery is an effective procedure for the treatment of OSA.^11^ The protocol was registered with Prospero (registration number: CRD42018114221) (Table 1).

**Table 1:** Guiding axes for the creation of the SR question.

**Population**	Adult individuals diagnosed with OSA (PSG)
**Intervention**	Orthognathic surgery (maxillomandibular advancement)
**Comparison**	Non-treatment (pre-op) & orthognathic surgery (post-op). NOTE: patients are their own controls
**Outcomes**	**Outcome 1:** apnea resolution (AHI < 5 events/h)
	**Outcome 2:** AHI < 15 events/h. NOTE: values greater than 15 are indicators of increased cardiovascular risk secondary to OSA
	**Outcome 3:** 50% reduction of the initial preoperative AHI value
	**Outcome 4:** preoperative AHI X postoperative AHI. Variable submitted to meta-analysis

### Study selection

Cohort studies with adult patients submitted to MMA for the treatment of OSA that contained pre- and postoperative polysomnography results were included in the review.

Studies in which patients underwent other surgical procedures concomitantly with MMA that could influence respiratory function and generate evaluation bias, such as nasal and pharyngeal surgeries, were excluded. Syndromic patients or those with cleft lip and palate were also excluded from this study, due to possible maxillomandibular anatomical alterations involving the UA, which these individuals normally have. Systematic reviews, reviews and case reports were also excluded.

### Data extraction

Two reviewers (PAKT and SATW) independently extracted data from the studies. Discrepancies at this stage were resolved by discussion and consensus. A form was used to extract the following information: type of study, number of patients, gender, mean age, pre- and postoperative Body Mass Index (BMI), postoperative time of follow-up, type of intervention, amount of advancement (maxillary and mandibular) and the pre- and postoperative AHI.

### Meta-analysis

Pre- and postoperative AHI was the variable susceptible to meta-analysis, carried out using Review Manager 5.3.5 (RevMan) program, developed by the Cochrane Collaboration.^12^

### Risk of bias assessment

To assess the risk of bias in the studies, the Modified Delphi technique tool was used, which consists of a questionnaire developed for the analysis of cohort studies, consisting of 18 questions with ‘yes’ or ‘no’ answers that generate a score from 0 to 18, where a number equal to or greater than 14 yes answers characterizes a study of acceptable methodological quality.^13^ Criteria such as clarity indicating the hypothesis and study objective, appropriate eligibility criteria, description of the therapeutic intervention, collection methodology, analysis and interpretation of results, time of follow-up and adverse events are part of the proposed analysis.

### Assessment of the quality of evidence

The quality of the evidence was assessed using the GRADE tool, developed by the Cochrane collaboration, that evaluates the type of study, number of study participants, presence, and severity of the risk of bias, estimation of the effect of the intervention, with a result of very low, low, moderate, or high quality.^14^

## Results

**Figure 1:**
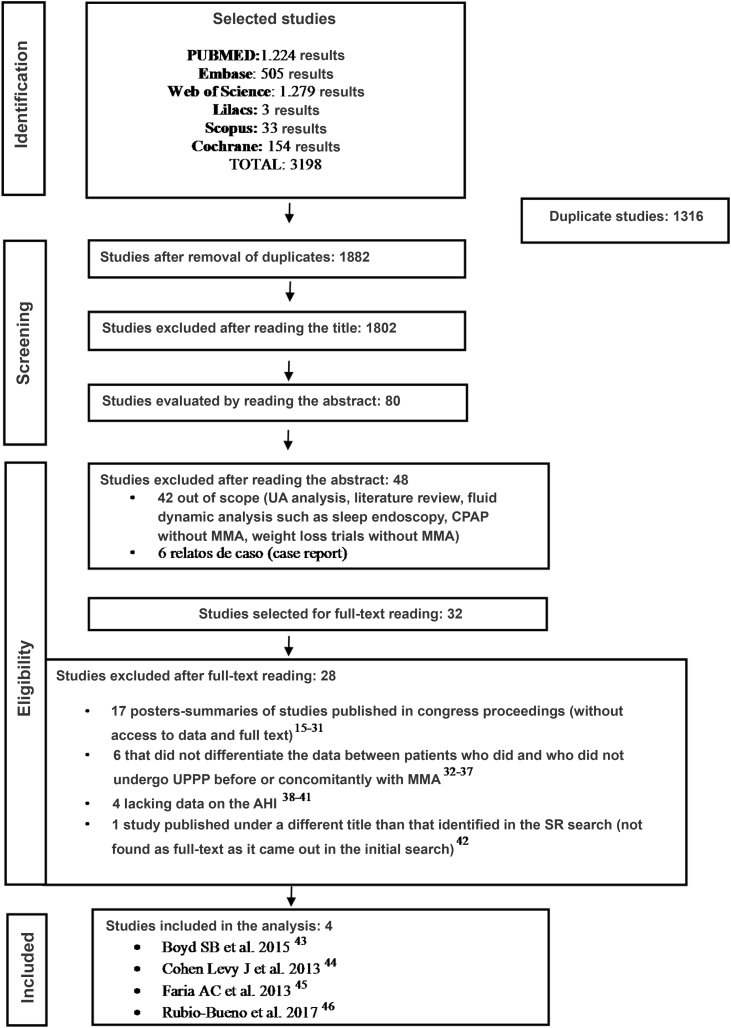
Systematic review flowchart. Source: adapted from PRISMA.

Of 1882 studies found in the literature search, 32 were selected for full-text reading. Of these 32 studies, 28 were excluded after applying the eligibility criteria, with four studies remaining for the final analysis (Fig. 1). All studies included for the meta-analysis were cohort/case series published between 2013 and 2017. No randomized controlled trials were found.

The total sample of the four studies included in this SR consisted of 83 adult individuals who underwent MMA for the treatment of OSA with pre- and postoperative PSG assessment. Of these, 68.68% were male and 31.32% were female. The overall average of the preoperative AHI was 40.81 ± 15.66. In the postoperative period, the overall average of the AHI was 8.33 ± 7.03, characterizing a mean percentage AHI reduction of 79.5%. Postoperative PSG assessments were performed after at least six months of post-surgical clinical follow-up. Only the study by Cohen-Levy et al. in 2013 did not mention the specific period in which the polysomnography assessments were performed postoperatively. The overall average of the preoperative BMI was 28.13 kg/m^2^ ± 4.17 and the postoperative average was 27.30 kg/m^2^ ± 3.91. The average amount of surgical maxillary advancement was 6.53 ± 2.74 mm, whereas the average mandibular advancement was 10.02 ± 3.29 mm (Table 2).

**Table 2:** Overall data from the total sample of studies included in the SR.

Sample	83 adult individuals submitted to MMA
Male sex	68.68%
Female sex	31.32%
Pre‒op AHI (overall average)	40.81 ± 15.66 (events/h)
Post‒op AHI (overall average)	8.33 ± 7.03 (events/h)
Mean reduction of AHI	79.5%
Pre‒op BMI (overall average)	28.13 kg/m^2^ ± 4.17 (overweight)
Post‒op BMI (overall average)	27.30 kg/m^2^ ± 3.91
Average amount of maxillary advancement	6.53 ± 2.74 mm
Average amount of mandibular advancement	10.02 ± 3.29 mm

All studies were assessed for their risk of bias by applying the Modified Delphi technique questionnaire, proposed by Moga et al. in 2012. Even though the studies passed the strict eligibility criteria proposed by the authors of this systematic review, the analysis showed an increased risk of bias in one of the four studies in the meta-analysis.^45^

**Figure 2:**
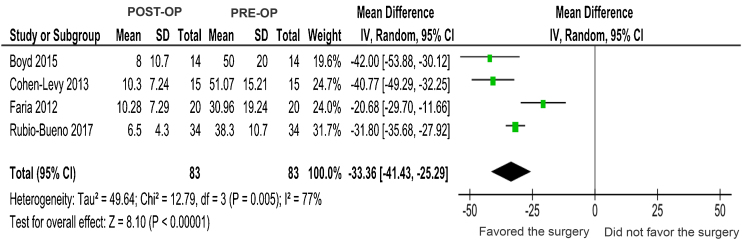
Meta-analysis: comparison of AHI pre- and post-MMA.

To evaluate the effect of the intervention, the meta-analysis of outcome 4 (pre- and postoperative AHI reduction) was performed. The four studies (Fig. 2) showed a significant difference in favor of surgical intervention, evaluating a total of 83 individuals with OSA submitted to MMA. When comparing the pre- and postoperative AHI, the mean difference was −33.36 (DM = −33.36, 95% CI −41.43 to −25.29, *p* < 0.00001), characterizing a mean reduction of 33.36 events of respiratory obstruction per hour of sleep, verified in the polysomnography test. Study heterogeneity was considered moderate/high due to the difference in the sample size of each study, considering that the composition of the groups was similar in terms of age, gender and BMI.

The assessment of the quality of evidence by GRADE resulted in very low quality. All studies started from a very low level of quality of evidence because they are non-controlled studies. The lack of a control group, longer time of follow-up and the small sample number of studies prevented the quality of the evidence from being high, even generating a higher heterogeneity. However, all studies pointed to an effect in favor of the surgical intervention (Table 3).

**Table 3:** Evaluation of the quality of evidence (GRADE).

**Summary of results:**
**Comparison of AHI pre- and post-MMA**
**Population:** adult individuals diagnosed with OSA
**Intervention:** MMA (maxilomandibular advancement)
**Comparison:** non-treatment (pre‒op) & MMA (post‒op)

Outcomes	Potential absolute effects* (95%CI)		Relative effect (95%CI)	N. of participants (studies)	Certainty of the evidence (GRADE)	Comments
	Risk with placebo	Risk with MMA X no MMA				
AHI	‒	DM **33.36 lower** (41.43 lower to 25.29 lower)	‒	83 (4 observational studies)	⨁◯◯◯ very low^a^, ^b^	
*The risk in the intervention group (and its 95% Confidence Interval) is based on the assumed risk of the comparison group and the **relative effect** of the intervention (and its 95% CI). **CI**, Confidence Interval; **DM**, difference of means						
**GRADE Working Group grades of evidence**						
**High certainty:** we are very confident that the true effect lies close to that of the estimate of the effect						
**Moderate certainty:** we are moderately confident in the effect estimate: the true effect is likely to be close to the estimate of the effect, but there is a possibility that it is substantially different						
**Low certainty:** our confidence in the effect estimate is limited: the true effect may be substantially different from the estimate of the effect						
**Very low certainty:** we have very little confidence in the effect estimate: the true effect is likely to be substantially different from the estimate of effect						

Explanations:

a All included studies are series of cases.

b Although the I^2^ = 77%, the individual results of each study had the same effect direction, favoring the intervention.

## Discussion

This systematic review evaluated the efficacy of MMA for the treatment of OSA. The main clinical criterion evaluated in this review was the AHI, found in the pre- and postoperative PSG tests. Complementary parameters such as age, BMI, time of follow-up or post-operative control, in addition to the amount of maxillomandibular advancement, were obtained to draw a profile of the individual with obstructive sleep apnea candidate to MMA.

The included studies did not show details about previous clinical treatments, except in the sample of the study by Boyd et al. in 2015, in which all patients were refractory to continuous positive airway pressure (CPAP) treatment and did not adapt to its continuous use. The mean age of the individuals was 45.34 ± 12.04 years, according to the data provided in the studies by Boyd et al. in 2015 and Rubio-Bueno et al. in 2017, with most patients being male (68.68%). The overall average of the preoperative AHI was 40.81 ± 15.66; characterizing the sample of this systematic review as having severe OSA. The OSA Severity classification consists of a scale that considers 0–5 events/h = normal; 5–15 events/h = mild; 15–30 events/h = moderate and over 30 events/h = severe.^1^ The postoperative overall mean AHI was 8.33 ± 7.03, which represented the conversion of severe OSA to cure or mild/moderate OSA. The overall mean BMI was 28.13 kg/m^2^ ± 4.17 preoperatively and 27.30 kg/m^2^ ± 3.91 postoperatively, with individuals being classified as overweight. There was no significant difference between the pre- and postoperative BMI values; therefore, there was no decrease in the AHI secondary to weight loss during the assessed period.

The studies did not distinctly classify patients’ facial and occlusal types. These data would be extremely important to better characterize the profile of patients with OSA normally submitted to maxillomandibular advancement. However, due to the high values of the amount of maxillary and mandibular advancement, it is possible to consider that the individuals had a convex profile associated with some degree of mandibular retrusion, since the average mandibular advancement was always greater than the maxillary advancement (average maxillary advancement: 6.53 ± 2.74; average mandibular advancement: 10.02 ± 3.29).

The studies in this SR showed a lack of consistent information about the performance and nature of the mentoplasty movement. Failure to detail this intervention, commonly associated with MMA, downgraded the level of evidence and increased the risk of bias.

The reduced time of follow-up was also a factor that did not allow conclusions to be drawn about MMA as a treatment of OSA in the long term, since aging tends to generate an increased risk of loss of muscle tone and a tendency to gain weight. Weight. Only the study by Boyd et al. in 2015 was accurate when describing the time of follow-up (6.6 ± 2.8 years). The other included studies did not inform the standard deviation of the time of follow-up, hindering a more precise analysis and increasing the risk of sample bias.

However, the average reduction in the AHI was 79.5%, which is a positive result in favor of MMA. Postoperative AHI values remained below the index considered for the highest cardiovascular risk (AHI < 15 events/h), supporting the role of surgery as an alternative therapeutic proposal to CPAP, still considered the first-line therapy for the treatment of OSA.

The evaluation of some of the outcomes proposed by the PICO strategy in this review was hindered by the lack of complete and individualized data for each patient in the samples. Only the studies by Boyd et al. in 2015 and Rubio-Bueno et al. in 2017 identified the percentage of individuals who were cured of OSA with an AHI index <5 events/h (46.7% and 52.94%, respectively). In the average of these two studies, 50.01% of the patients were cured of OSAS (AHI < 5 events/h) and 89.89% of the patients attained AHI values <15 events/h, being outside the risk area for cardiovascular events resulting from OSA.

The meta-analysis of the four studies included in the SR showed a statistically significant difference in favor of the surgical intervention, evaluating a total of 83 individuals with OSA submitted to MMA. The average difference between the pre- and postoperative AHI was −33.36 events/h (−41.43, −25.29). When synthesizing the clinical data, it was possible to conclude that MMA was effective in reducing the AHI in all 4 studies included in the SR, with an average reduction of 79.5% in the postoperative period. These data are consistent in favor of the intervention and indicate the efficacy of the MMA effect on the treatment of OSA. However, as they are cohort studies, they are classified as having low quality of evidence at the outset, according to the GRADE method.

The therapeutic success observed in MMA is justified by the volumetric increase of the upper airways, since the pathophysiology is associated with diffuse narrowing of the pharynx, including the palatine region, lateral wall and base of the tongue. MMA increases the pharyngeal caliber by direct traction on the mandible, maxilla, and associated soft tissue structures, decreasing airway collapse during sleep.

However, this systematic review was able to demonstrate important and positive results of MMA as a treatment for OSA and distinguished itself by establishing strict criteria for the inclusion of studies, avoiding factors that would increase the risk of bias.

The predominant sample profile comprised male gender individuals, in the fourth or fifth decades of life, overweight, with some degree of mandibular retrusion associated with a convex facial profile, with severe OSA and possibly refractory or non-responsive to non-surgical clinical treatment.

## Conclusions

According to the results of this SR, it was possible to conclude that MMA was an effective procedure in the treatment of OSA and achieved an average reduction of 79.5% in the AHI in the 83 individuals of the sample. The meta-analysis comparing the pre- and postoperative AHI in individuals submitted to MMA was favorable to the procedure in all four included studies. The recommendation in favor of the intervention is weak due to the low quality of the evidence. However, there is consistency favoring the effect of MMA for the treatment of OSA.

## Conflicts of interest

The authors declare no conflicts of interest.

## References

[1] Berry R.B., Budhiraja R., Gottlieb D.J., Gozal D., Iber C., Kapur V.K. (2012). Rules for scoring respiratory events in sleep: update of the 2007 AASM manual for the scoring of sleep and associated events. Deliberations of the sleep apnea definitions task force of the American Academy of Sleep Medicine. J Clin Sleep Med.

[2] Moyer C.A., Sonnad S.S., Garetz S.L., Helman J.I., Chervin R.D. (2001). Quality of life in obstructive sleep apnea: a systematic review of the literature. Sleep Med.

[3] Reimer M.A., Flemons W.W. (2003). Quality of life in sleep disorders. Sleep Med Rev.

[4] Young T., Peppard P.E., Gottlieb D.J. (2002). Epidemiology of obstructive sleep apnea: a population health perspective. Am J Respir Crit Care Med.

[5] Weaver T.E., Grunstein R.R. (2008). Adherence to continuous positive airway pressure therapy: the challenge to effective treatment. Proc Am Thorac Soc.

[6] Brander P.E., Soirinsuo M., Lohela P. (1999). Nasopharyngeal symptoms in patients with obstructive sleep apnea syndrome. Effect of nasal CPAP treatment. Respiration.

[7] Raffaini M., Pisani C. (2013). Clinical and cone-beam computed tomography evaluation of the three-dimensional increase in pharyngeal airway space following maxillo-mandibular rotation-advancement for Class II-correction in patients without sleep apnoea (OSA). J Craniomaxillofac Surg.

[8] Abramson Z., Susarla S.M., Lawler M., Bouchard C., Troulis M., Kaban L.B. (2011). Three-dimensional computed tomographic airway analysis of patients with obstructive sleep apnea treated by maxillomandibular advancement. J Oral Maxillofac Surg.

[9] Fairburn S.C., Waite P.D., Vilos G., Harding S.M., Bernreuter W., Cure J. (2007). Three-dimensional changes in upper airways of patients with obstructive sleep apnea following maxillomandibular advancement. J Oral Maxillofac Surg.

[10] Hernández-Alfaro F., Guijarro-Martínez R., Mareque-Bueno J. (2011). Effect of mono and bimaxillary advancement on pharyngeal airway volume: cone-beam computed tomography evaluation. J Oral Maxillofac Surg.

[11] Galvão T.F., Pansani T.S.A., Harrad D. (2015). Principais itens para relatar Revisões sistemáticas e Meta-análises: A recomendação PRISMA. Epidemiologia e Serviços de Saúde.

[12] The Nordic Cochrane Centre TCC (2014).

[13] Moga C., Guo B., Schopflocher D., Harstall C. (2012).

[14] Guyatt G.H., Oxman A.D., Vist G.E., Kunz R., Falck-Ytter Y., Alonso-Coello P. (2008). GRADE: an emerging consensus on rating quality of evidence and strength of recommendations. BMJ.

[45] Faria A.C., da Silva-Junior S.N., Garcia L.V., dos Santos A.C., Fernandes M.R., de Mello-Filho F.V. (2013). Volumetric analysis of the pharynx in patients with obstructive sleep apnea (OSA) treated with maxillomandibular advancement (MMA). Sleep Breath.

